# The WWW of the World Wide Web: Who, What, and Why?

**DOI:** 10.2196/jmir.4.1.e4

**Published:** 2002-02-18

**Authors:** John Powell, Aileen Clarke

**Affiliations:** ^1^Health Services Research UnitLondon School of Hygiene and Tropical MedicineKeppel StreetLondon WC1E 7HTUK

Current estimates suggest that 25 million people in the UK have access to the World Wide Web, and 14 million use it regularly [1]. Worldwide over 500 million people have logged on [2]. They have access to over 3 billion Web documents [3], and at least 2% of Web sites are health related [4]. Indeed, accessing health information is one of the commonest reasons for going on-line: surveys show that 50% to 75% of World Wide Web users have used it to look for health information [5,6,7], and those who do so access such information over 3 times a month [5]. In December 2001 the NHS Direct consumer health information Web site (www.nhsdirect.nhs.uk) dealt with 5.2 million hits from 171900 visitors [8].

Physicians are increasingly experiencing patients bringing Internet printouts to the consultation, although estimates of the frequency of this occurrence vary from 1-2% [9], to 58% [10], to over 70% [11]. The low prevalence of Internet-savvy patients of only 1-2% in the study by Potts and Wyatt [9], published in this issue of the *Journal of Medical Internet Research*, is surprising given the findings from consumer surveys on the frequency of accessing online health information that are cited above. Potts and Wyatt used a cross-sectional survey method asking respondents to retrospectively estimate the number of their patients who had used the Internet for health information in the past month. It is possible that recall bias may have led to an underestimate, but it is also likely that not all patients who consult the Internet reveal this to their doctor. The potential impact of the wide availability of online health information on the practitioner-patient relationship has been debated [12,13]. The Internet is a key influence in changing the balance of (knowledge) power between health care professionals and the public, empowering patients to become more involved in health care decision making and contributing to the deprofessionalization of medicine. Empirical research is beginning to investigate this impact [9,14].

Much of the limited evidence as to who the consumers of Internet health information are and what they are looking for comes from United States market-research surveys and Web-usage statistics, both quantifying the numbers of users and types of information accessed. Women are more likely than men to seek health care information on-line, and the highest proportion of usage is in those between 30 and 64 years old [15]. Use of the Internet for health information declines with age [16,17]. Despite the much-discussed "digital divide" between the higher-income, more-educated "have-nets" and the lower-income, less-educated "have-nots," there is no evidence of differences in health-information seeking by income group once they have on-line access [18,19].

A 1999 Harris Poll of 2000 US adults found that mental health issues dominated the most popular on-line health topics, with depression, bipolar disorder, and anxiety problems accounting for 42% of the use of the Web to find health information [20]. Further work to investigate which health topics are most frequently accessed on-line will be valuable. Most on-line health seekers are looking for a specific answer to a specific health question, and start by submitting a topic to a general search engine [21]. Far fewer users go to health portals or direct to a specific health site, and in general, users do not simply browse for health-related information [22]. Most users research specific health issues that are currently affecting a friend, relative, or themselves, frequently in connection with a visit to their doctor [15]. Few use health sites to communicate with health services, purchase pharmaceuticals, or participate in health-related chat-room discussions [15]. However, the majority of US users report a desire for more on-line interaction with their doctors, including e-mail consultations and reminders [23].

The research in this area is notable for a relative lack of qualitative work exploring the reasons behind on-line information seeking and the attitudes and behavior of health users towards the World Wide Web. Sociological work has led to a better understanding of the process of help-seeking behavior, but this work now needs to be updated to take into account the use of the Internet by patients and caregivers.

Users report valuing the convenience, anonymity, and volume of online information [15]. It is likely that individuals will use the Web at different points in the trajectory of illness and health care. The California Healthcare Foundation has attempted to categorize 3 types of user - the well; the newly diagnosed; and the chronically ill and their caregivers [24]. The well group carries out episodic searching for information relating to short-term medical conditions, pregnancy, and prevention issues. The newly diagnosed carries out very intensive searching for specific information, valuing the ease of access and broad range of information. The chronically ill and their caregivers carry out regular searching for information related to new treatments, nutrition advice, and alternative therapies. In addition, the latter 2 groups both value and use on-line communities and chat rooms. Several studies have shown the importance of the World Wide Web in providing social support, particularly to groups with chronic health problems such as diabetes patients [25. 26] or individuals with HIV [27].

It is likely that much of what is required from online information will be similar to that required from more-traditional routes: clear, well-presented information, with advice on further sources. However, there may well also be particular advantages that can be gained from the interactivity, personalization, and creative ways of managing knowledge that the Internet provides. For example, preliminary work suggests that the Internet may be an effective means of delivering psychological therapies [28].

In an era of user involvement, consumer empowerment, and the wide dissemination of information on health and health services, it is important that we identify who the consumers of online health information are, what their information needs are, and understand why and how they seek information online. This will enable information to be provided in ways that will have benefits from the worldwide to the individual level, and will inform current debates over the quantity and quality of information provision and issues of privacy and access.
